# Supplementary material for: EHMT2 epigenetically suppresses Wnt signaling and is a potential target in embryonal rhabdomyosarcoma
Source: eLife. 2020 Nov 30;9:e57683. doi: 10.7554/eLife.57683 (PMC7728445; doi:10.7554/eLife.57683)
Supplement: Source data 1. [file elife-57683-data1.pdf]

Figure 1C

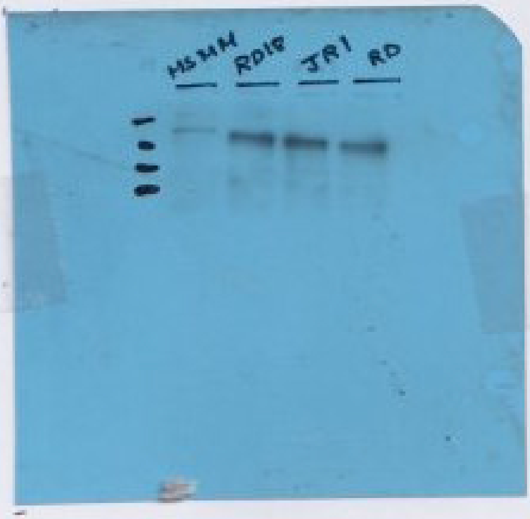

Primary Ab: G9a
dilution: 1:300
Secondary Ab: Rabbit
dilution: 1:5000

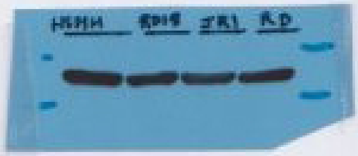

Primary Ab: β-actin
dilution: 1:10000
Secondary Ab:
dilution: 1:5000

Figure 2A

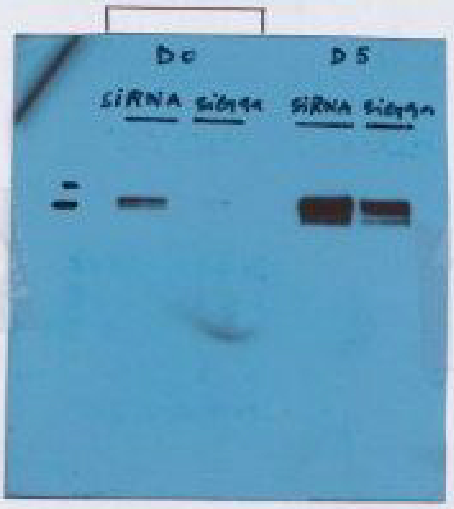

Primary Ab : G9a
Dilution : 1:800
Secondary Ab : Rabbit
Dilution : 1:5000

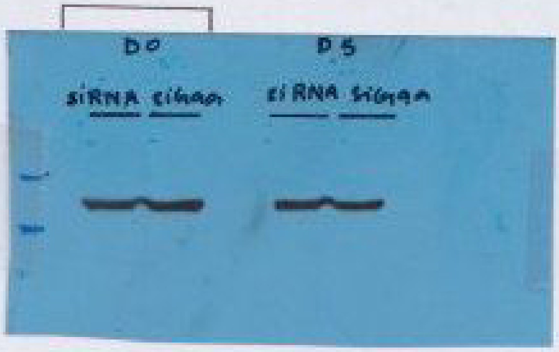

Primary Ab : β-actin
Dilution : 1:10000
Secondary Ab : Mouse
Dilution : 1:5000

Figure 2B

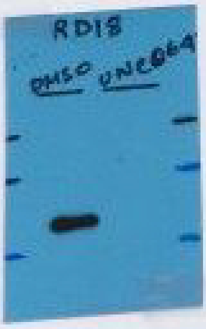

Primary Ab: H3K9me2
Dilution : 1:1000
Secondary Ab : Rabbit
Dilution : 1:5000

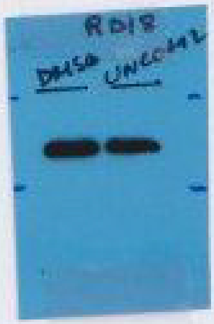

Primary Ab: H3
Dilution: 1:10000
Secondary Ab : Rabbit
Dilution: 1:5000

Figure 2C (siRNA, siG9a)

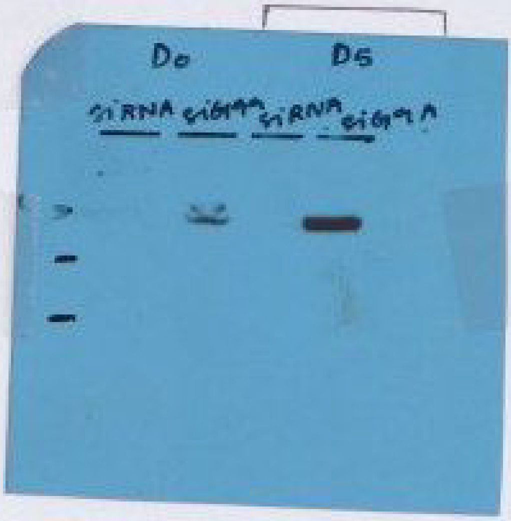

Primary Ab: MHC

Dilution: 1:300

Secondary Ab: Mouse

Dilution: 1:5000

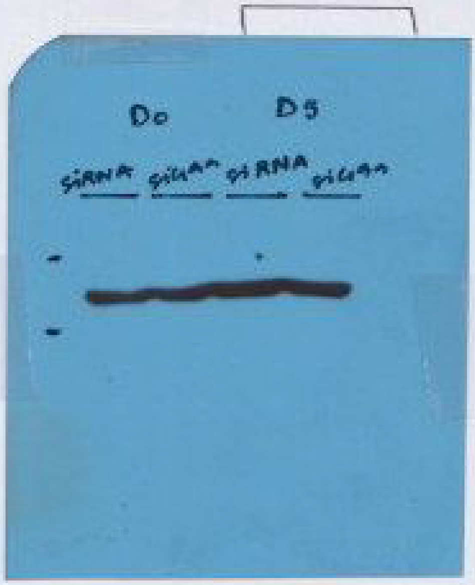

Primary Ab: β-actin

Dilution: 1:10000

Secondary Ab: Mouse

Dilution: 1:5000

Figure 2C (DMSO, UNC0642)

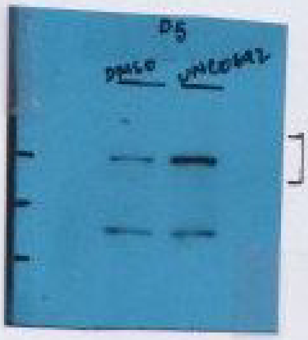

Primary Ab : MHC

Dilution : 1:300

Secondary Ab : Mouse

Dilution : 1:5000

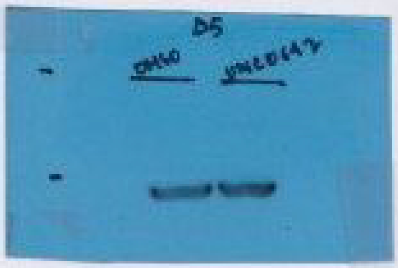

Primary Ab : β-actin

Dilution : 1:10000

Secondary Ab: Mouse

Dilution : 1:5000

Figure 3F, G

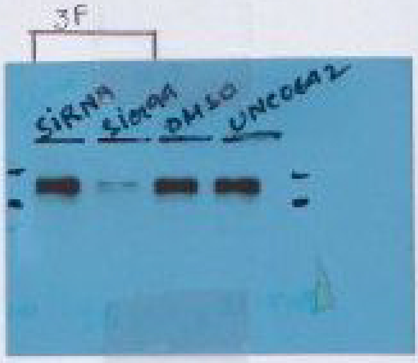

Primary Ab : G9a
Dilution : 1 : 800
Secondary Ab : Rabbit
Dilution : 1 : 5000

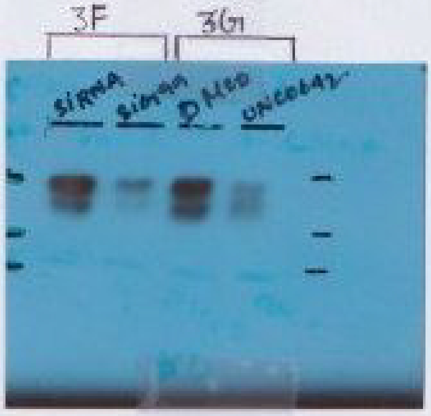

Primary Ab : DKK1
Dilution : 1 : 800
Secondary Ab : Mouse
Dilution : 1 : 5000

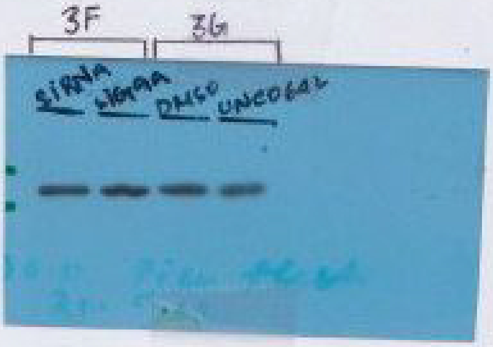

Primary Ab : β-actin
Dilution : 1 : 10 000
Secondary Ab : Mouse
Dilution : 1 : 5000

Figure 3H

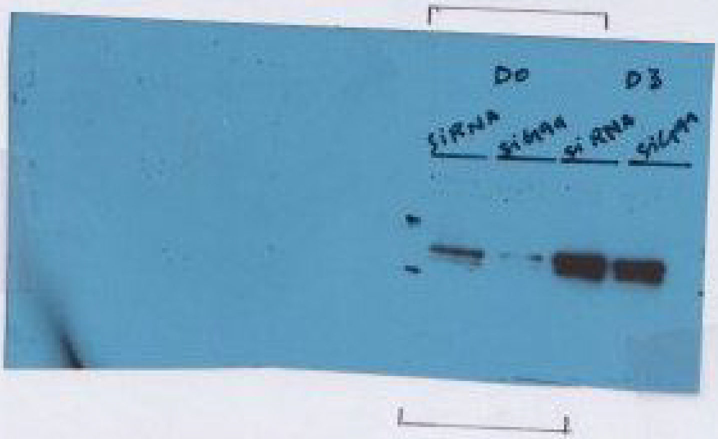

Primary Ab: G9a

Dilution: 1:300

Secondary Ab: Rabbit

Dilution: 1:5000

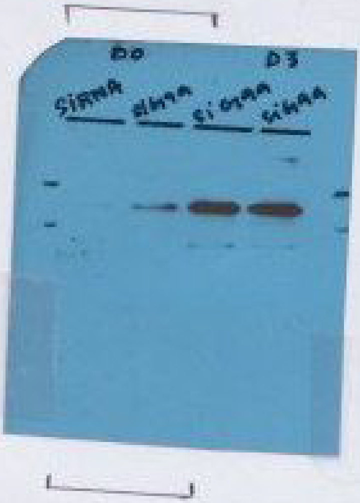

Primary Ab: A-β-catenin

Dilution: 1:500

Secondary Ab: Mouse

Dilution: 1:5000

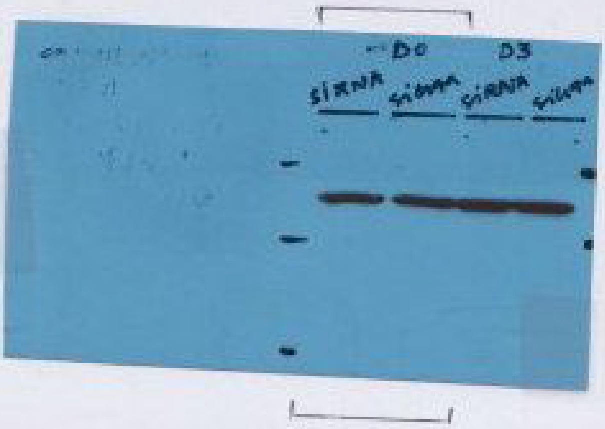

Primary Ab: β-actin

Dilution: 1:10000

Secondary Ab: Mouse

Dilution: 1:5000

Figure 3I

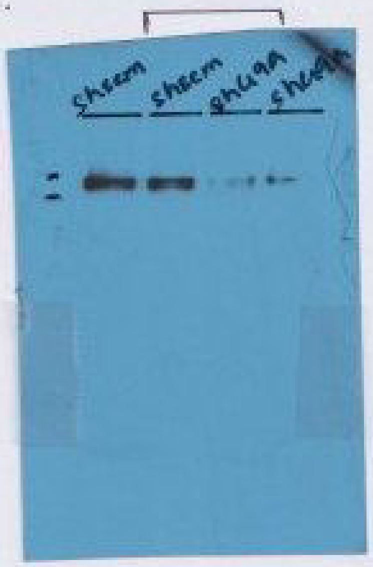

Primary Ab: Gαq

Dilution: 1:300

Secondary Ab: Rabbit

Dilution: 1:5000

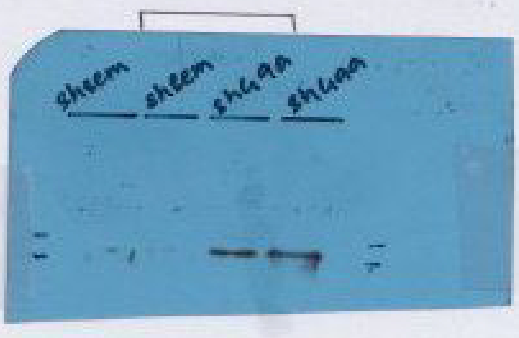

Primary Ab: A-β-catenin

Dilution: 1:500

Secondary Ab: Mouse

Dilution: 1:5000

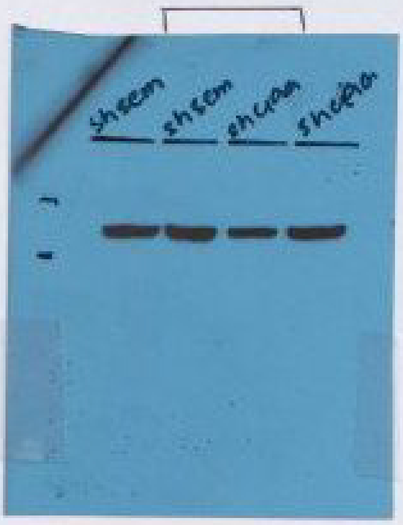

Primary Ab: β-actin

Dilution: 1:10000

Secondary Ab: Mouse

Dilution: 1:5000

Figure 3J

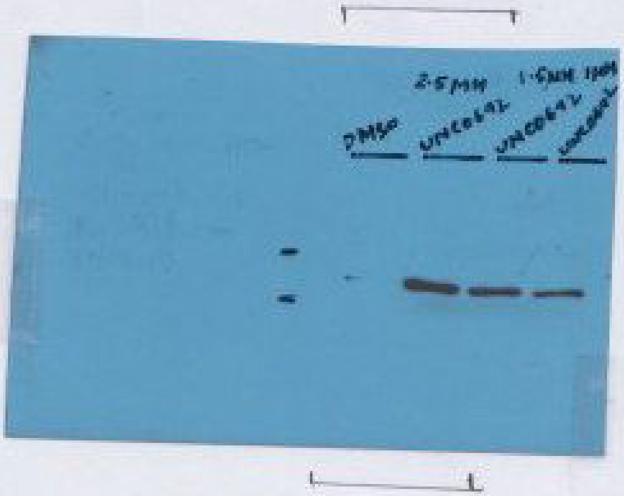

Primary Ab: A-β-catenin
Dilution: 1:500
Secondary Ab: Mouse
Dilution: 1:5000

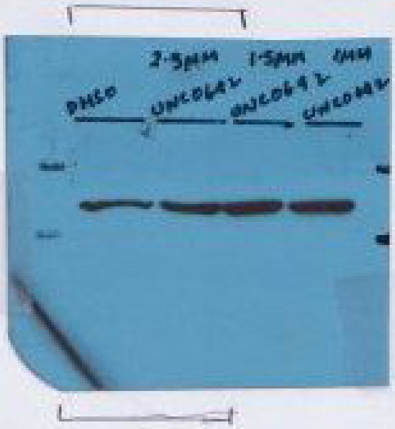

Primary Ab: β-actin
Dilution: 1:10000
Secondary Ab: Mouse
Dilution: 1:5000

Figure 4F

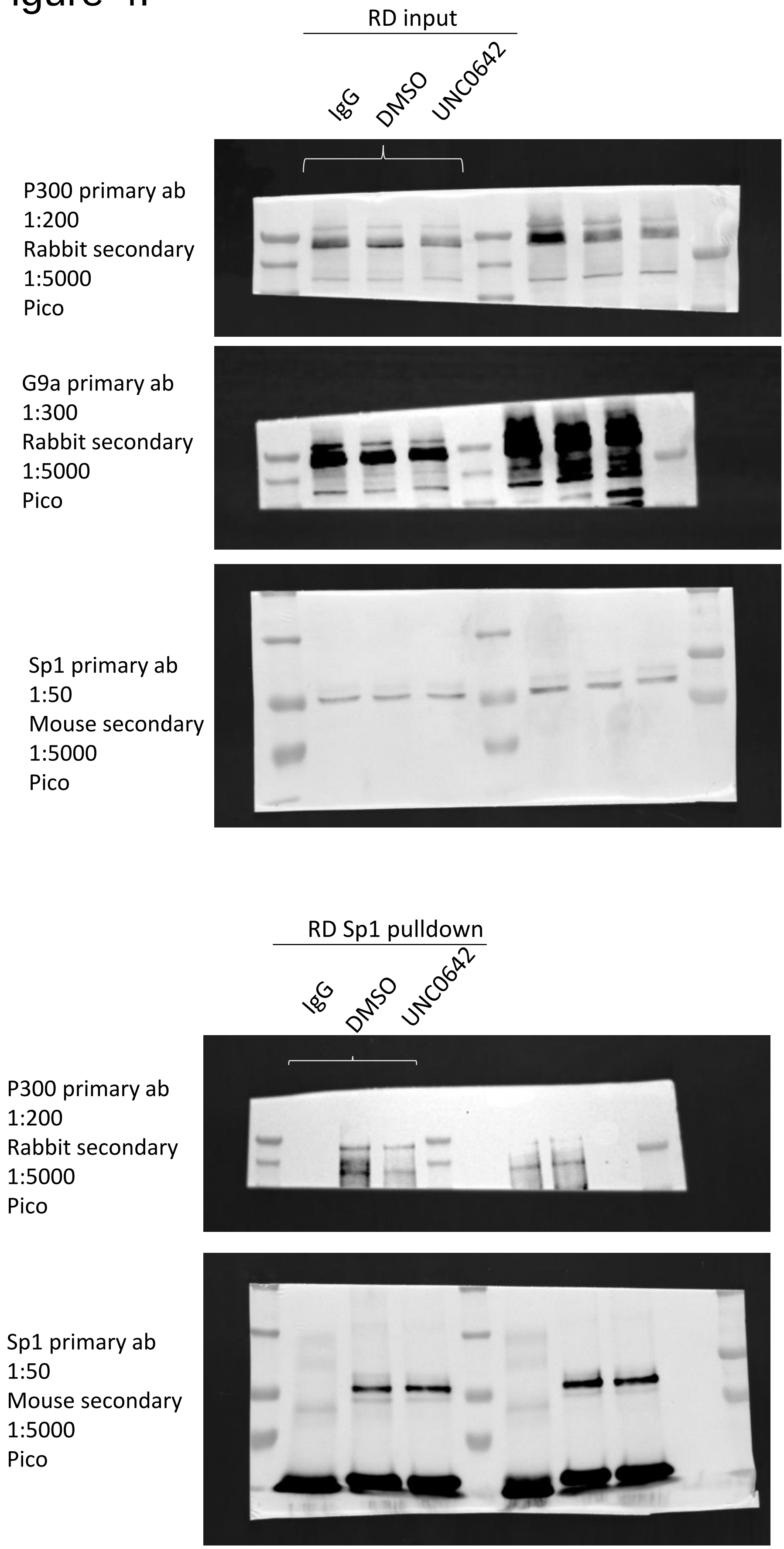
RD input
IgG
DMSO
UNC0642
P300 primary ab
1:200
Rabbit secondary
1:5000
Pico
G9a primary ab
1:300
Rabbit secondary
1:5000
Pico
Sp1 primary ab
1:50
Mouse secondary
1:5000
Pico
RD Sp1 pulldown
IgG
DMSO
UNC0642
P300 primary ab
1:200
Rabbit secondary
1:5000
Pico
Sp1 primary ab
1:50
Mouse secondary
1:5000
Pico

Figure 5B

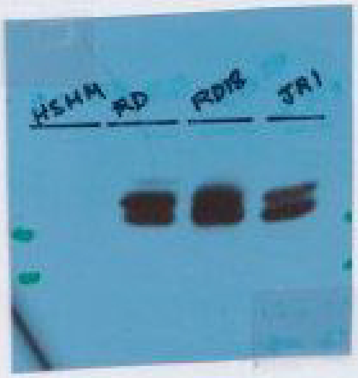

Primary Ab: DUK1
Dilution: 1:500
Secondary Ab: Mouse
Dilution: 1:5000

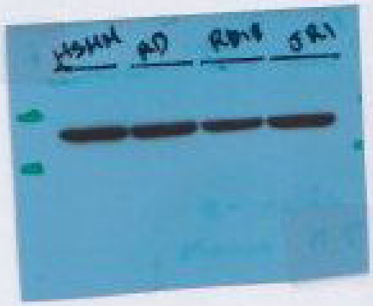

Primary Ab: β-actin
Dilution: 1:10000
Secondary Ab: Mouse
Dilution: 1:5000

Figure 5C

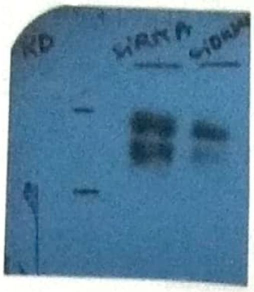

Primary Ab : DKK1
Dilution : 1 : 300
Secondary Ab : Mouse
Dilution : 1:5000

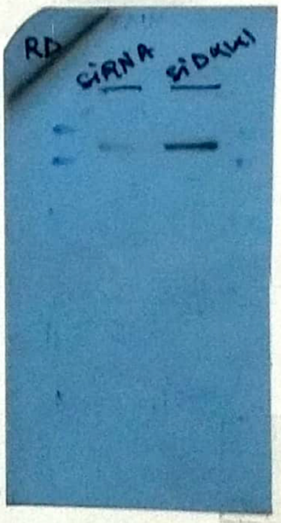

Primary Ab : A-β-catenin
Dilution : 1:500
Secondary Ab : Mouse
Dilution : 1:5000

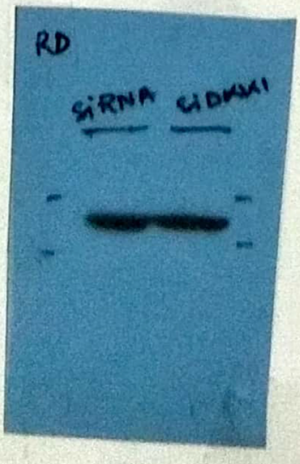

Primary Ab : β-actin
Dilution : 1:10000
Secondary Ab : Mouse
Dilution:1:5000

Figure 5D

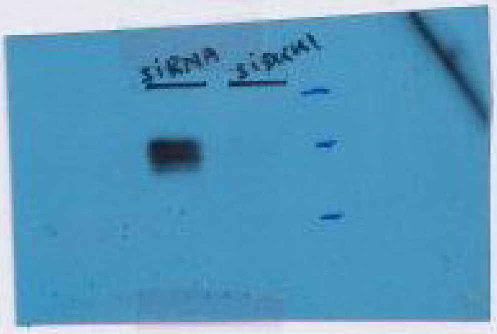

Primary Ab: DKK1
Dilution: 1:300
Secondary Ab: Mouse
Dilution: 1:5000

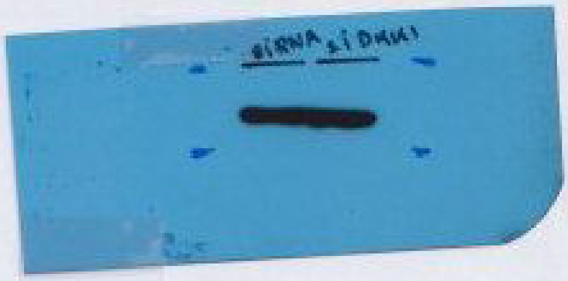

Primary Ab: β-actin
Dilution: 1:10000
Secondary Ab: Mouse
Dilution: 1:5000

Figure 5E

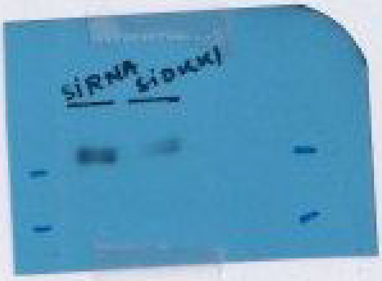

Primary Ab : DKK1
Dilution : 1:300
Secondary Ab : Mouse
Dilution : 1:5000

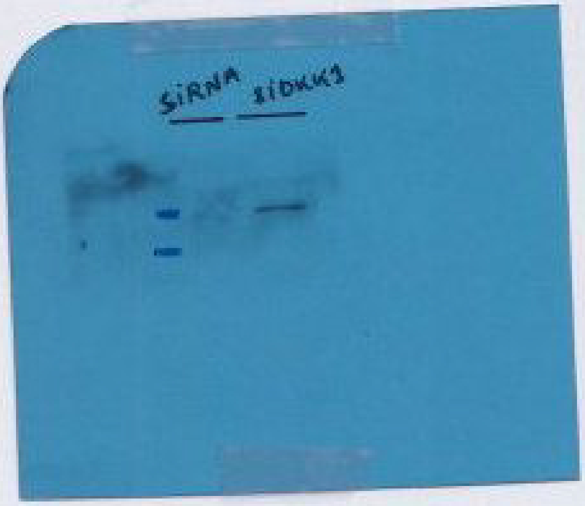

Primary Ab : MHC
Dilution : 1:300
Secondary Ab : Mouse
Dilution : 1:5000

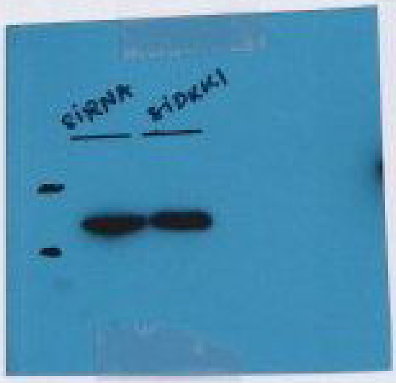

Primary Ab : β-actin
Dilution : 1:10000
Secondary Ab : Mouse
Dilution : 1:5000

Figure 5F

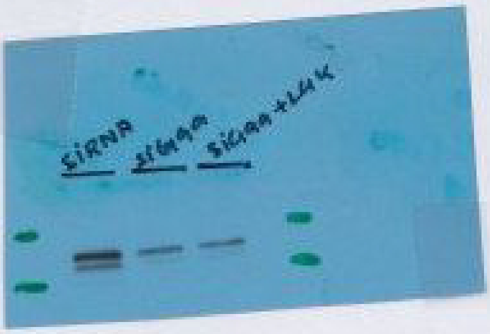

Primary Ab: Gqa
Dilution: 1:300
Secondary Ab: Rabbit
Dilution: 1:5000

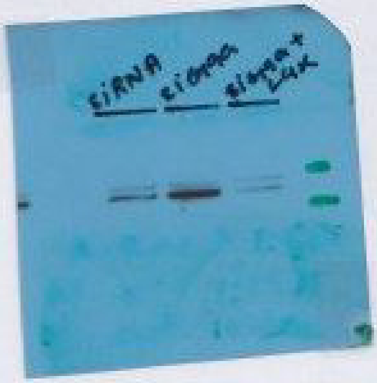

Primary Ab: A-β-catenin
Dilution: 1:500
Secondary Ab: Mouse
Dilution: 1:5000

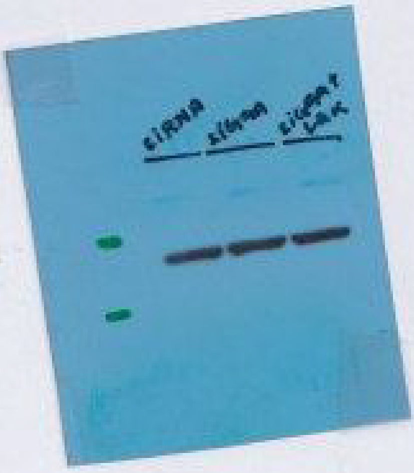

Primary Ab: β-actin
Dilution: 1:10 000
Secondary Ab: Mouse
Dilution: 1:5000

Figure 5G

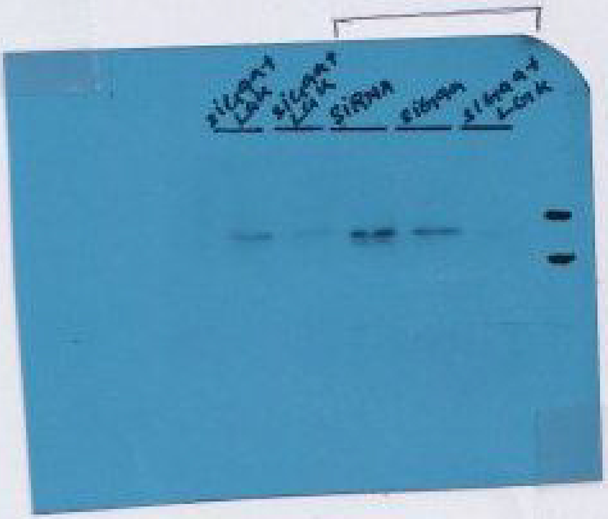

Primary Ab: Gaa
Dilution: 1:300
Secondary Ab: Rabbit
Dilution: 1:5000

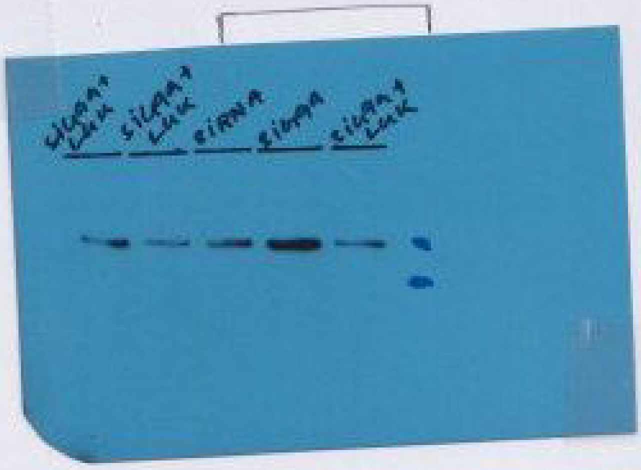

Primary Ab: A-β-catenin
Dilution: 1:500
Secondary Ab: Mouse
Dilution: 1:5000

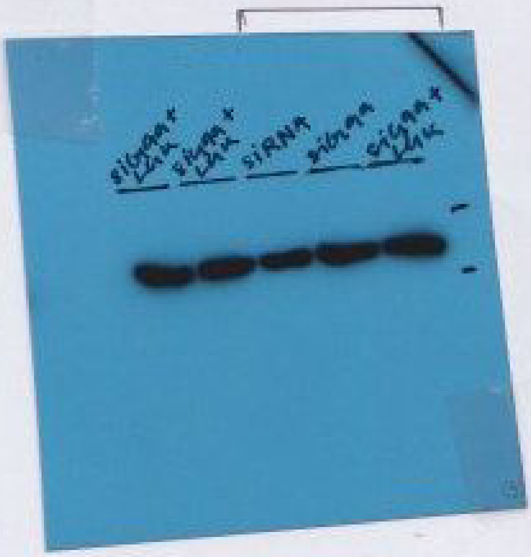

Primary Ab: β-actin
Dilution: 1:10000
Secondary Ab: Mouse
Dilution: 1:5000
