# Supplementary material for: EHMT2 epigenetically suppresses Wnt signaling and is a potential target in embryonal rhabdomyosarcoma
Source: eLife. 2020 Nov 30;9:e57683. doi: 10.7554/eLife.57683 (PMC7728445; doi:10.7554/eLife.57683)
Supplement: Transparent reporting form [file elife-57683-transrepform.docx]

***eLife’s* transparent reporting form**

We encourage authors to provide detailed information *within their submission* to facilitate the interpretation and replication of experiments. Authors can upload supporting documentation to indicate the use of appropriate reporting guidelines for health-related research (see [EQUATOR Network](http://www.equator-network.org/%20)), life science research (see the [BioSharing Information Resource](https://biosharing.org/)), or the [ARRIVE guidelines](http://www.plosbiology.org/article/info:doi/10.1371/journal.pbio.1000412) for reporting work involving animal research. Where applicable, authors should refer to any relevant reporting standards documents in this form.

If you have any questions, please consult our Journal Policies and/or contact us: [editorial@elifesciences.org](mailto:editorial@elifesciences.org).

**Sample-size estimation**

- You should state whether an appropriate sample size was computed when the study was being designed
- You should state the statistical method of sample size computation and any required assumptions
- If no explicit power analysis was used, you should describe how you decided what sample (replicate) size (number) to use

Please outline where this information can be found within the submission (e.g., sections or figure legends), or explain why this information doesn’t apply to your submission:

For our in vivo studies in Figure 6 the sample size of 10 per group was decided based on previous on alveolar rhabdomyosarcoma from our lab (Bhat et al, 2019).

**Replicates**

- You should report how often each experiment was performed
- You should include a definition of biological versus technical replication
- The data obtained should be provided and sufficient information should be provided to indicate the number of independent biological and/or technical replicates
- If you encountered any outliers, you should describe how these were handled
- Criteria for exclusion/inclusion of data should be clearly stated
- High-throughput sequence data should be uploaded before submission, with a private link for reviewers provided (these are available from both GEO and ArrayExpress)

Please outline where this information can be found within the submission (e.g., sections or figure legends), or explain why this information doesn’t apply to your submission:

For all experiments, information about biological and technical replicates can be found in the figure legends. The scatter plots in all figures show a plot of all individual data points (technical replicates and biological replicates). All error bars indicate standard error of mean of at least three biological replicates. Bar graphs in Figure 4A and in Supplemental Figure 3C-J and in Supplemental Figure 4A-D denotes standard deviation.

**Statistical reporting**

- Statistical analysis methods should be described and justified
- Raw data should be presented in figures whenever informative to do so (typically when N per group is less than 10)
- For each experiment, you should identify the statistical tests used, exact values of N, definitions of center, methods of multiple test correction, and dispersion and precision measures (e.g., mean, median, SD, SEM, confidence intervals; and, for the major substantive results, a measure of effect size (e.g., Pearson's r, Cohen's d)
- Report exact p-values wherever possible alongside the summary statistics and 95% confidence intervals. These should be reported for all key questions and not only when the p-value is less than 0.05.

Please outline where this information can be found within the submission (e.g., sections or figure legends), or explain why this information doesn’t apply to your submission:

All information has been provided in figure legends. Statistical significance was calculated by unpaired two-tailed t test for all graphs. This statistical analysis was chosen because it is an accepted method for testing a null hypothesis to determine whether the means of two samples are statistically significant.

For the graphs in Figure 6A and C, statistical significance was calculated using repeated –measure two-way ANOVA. This statistical analysis was chosen because it helps us determine how a response is affected by two factors: one factor being the two different treatment groups, and the other factor which is measured at repeated intervals.

P value summaries can be found in each respective figure legends. Exact values of statistical analyses, including P-values, can be found in Table 1 and 2 (Source Data).

(For large datasets, or papers with a very large number of statistical tests, you may upload a single table file with tests, Ns, etc., with reference to sections in the manuscript.)

**Group allocation**

- Indicate how samples were allocated into experimental groups (in the case of clinical studies, please specify allocation to treatment method); if randomization was used, please also state if restricted randomization was applied
- Indicate if masking was used during group allocation, data collection and/or data analysis

Please outline where this information can be found within the submission (e.g., sections or figure legends), or explain why this information doesn’t apply to your submission:

Mice were randomly put in experimental groups described in figure 6. No other criteria were applied.

**Additional data files (“source data”)**

- We encourage you to upload relevant additional data files, such as numerical data that are represented as a graph in a figure, or as a summary table
- Where provided, these should be in the most useful format, and they can be uploaded as “Source data” files linked to a main figure or table
- Include model definition files including the full list of parameters used
- Include code used for data analysis (e.g., R, MatLab)
- Avoid stating that data files are “available upon request”

Please indicate the figures or tables for which source data files have been provided:

Figures 1-6.
